# Prediction of treatment responsiveness to home-based transcranial photobiomodulation (tPBM) intervention for cognitive decline using fNIRS concurrently recorded during tPBM

**DOI:** 10.3389/fnagi.2026.1716502

**Published:** 2026-02-12

**Authors:** Minyoung Chun, Kyeonggu Lee, Bori Jung, Yunsu Kim, Chaeyeon Yang, JongKwan Choi, Jihyun Cha, Seung-Hwan Lee, Chang-Hwan Im

**Affiliations:** 1 Department of Electronic Engineering, Hanyang University, Seoul, Republic of Korea; 2 Department of Psychiatry, Inje University Ilsan Paik Hospital, Goyang, Republic of Korea; 3 Clinical Emotion and Cognition Research Laboratory, Inje University Ilsan Paik Hospital, Goyang, Republic of Korea; 4 OBELAB Inc., Seoul, Republic of Korea; 5 Department of Biomedical Engineering, Hanyang University, Seoul, Republic of Korea

**Keywords:** functional connectivity, functional near-infrared spectroscopy, graph theory, mild cognitive impairment, subjective cognitive decline, transcranial photobiomodulation

## Abstract

**Introduction:**

Transcranial Photobiomodulation (tPBM) has attracted growing interest as an intervention to mitigate cognitive decline in older adults. However, some individuals do not respond to tPBM. This study explored the feasibility of predicting treatment responsiveness using functional near-infrared spectroscopy (fNIRS) recorded during therapy with a device integrating tPBM and fNIRS.

**Methods:**

Twenty-nine participants with cognitive decline underwent 12-week home-based tPBM intervention with concurrent fNIRS acquisition. Notably, fNIRS data were collected using the existing tPBM light sources, without additional hardware. After termination of the intervention, patients were classified as responders or non-responders based on changes in the global cognitive score (ΔGCS), which reflects multiple cognitive domains. Fourteen participants were classified as responders and 15 as non-responders. fNIRS data from the initial 15 trials were segmented into 5 periods. Linear regression analysis was performed to evaluate the changes in graph-theoretical indices calculated from the functional connectivity analysis of fNIRS and their relationship with ΔGCS. Participants with regression values below a designated threshold were predicted as non-responders.

**Results:**

Significant negative correlations between ΔGCS and the changes in graph-theoretical indices were observed in periods 3–5. Participants with regression values below a designated threshold were predicted as non-responders. In total, 13 participants were identified as non-responders, with 11 confirmed as non-responders after tPBM therapy.

**Conclusion:**

We explored the feasibility of applying graph-theoretical network analysis to fNIRS data for the early identification of non-responders to tPBM treatment before its completion. This novel approach can potentially enhance treatment efficacy by allowing for timely treatment planning.

## 1 Introduction

With a growing older population, the increasing prevalence of neurodegenerative disorders has become a major concern. Subjective cognitive decline (SCD) refers to a perceived decline in cognitive function without objective evidence of neuropsychological deficits (Rabin et al., 2017). Although SCD does not significantly affect daily life, it holds substantial clinical importance as a potential preclinical stage of mild cognitive impairment (MCI) and Alzheimer’s disease (AD) (Jessen et al., 2020; Jessen et al., 2014). Specifically, 26.6% of patients with SCD progress to MCI, a syndrome characterized by an objective decline in cognitive abilities relative to expectations based on age and educational level (Jessen et al., 2020; Lee et al., 2022; Mitchell et al., 2014). Moreover, both SCD and MCI are associated with an increased risk of conversion to AD, with evidence suggesting that these conditions may represent early manifestations of preclinical AD (Gauthier et al., 2006; Jessen et al., 2014). Therefore, timely intervention during the early stages of cognitive decline is crucial to preserve brain health and reduce the risk of progression to AD (Eshkoor et al., 2015; Jessen et al., 2020).

Pharmacological therapy remains the most common intervention for cognitive decline. However, achieving effective outcomes from pharmacological interventions in older adults with cognitive decline is challenging (Kasper et al., 2020; Kim et al., 2021). Moreover, these treatments carry the risk of multiple side effects (Doody et al., 2009; Petersen et al., 2005) and are often difficult to administer to individuals with minimal clinical symptoms, complicating the identification of specific therapeutic targets (Smart et al., 2017). In contrast, non-invasive neuromodulation treatments can target brain regions of interest with minimal side effects and are well-tolerated by patients (Mosilhy et al., 2022). Consequently, various non-invasive neuromodulation techniques are being actively investigated as promising alternative treatment options for older adults with cognitive decline (Chen J. et al., 2020; Gomes et al., 2019; Jones et al., 2023; Kim et al., 2021; Nardone et al., 2014; Vaque-Alcazar et al., 2021).

Among noninvasive neuromodulation techniques, transcranial photobiomodulation (tPBM) has gained significant attention as a safe and effective modality for enhancing cognitive functions in individuals with various neuropsychiatric disorders. In tPBM, red (600–670 nm) and near-infrared (800–1,100 nm) lights are projected through the scalp, targeting brain regions associated with cognitive processes. Vargas et al. (Vargas et al., 2017) demonstrated that participants with SCD showed significant improvements in sustained attention and working memory following 5-week tPBM treatment. Other studies have reported that tPBM effectively enhances cognitive function in patients with MCI (Chan et al., 2021; Cheung et al., 2022) and dementia (Chao, 2019; Nizamutdinov et al., 2021). However, similar to other noninvasive neuromodulation techniques, the efficacy of tPBM is inconsistent across patients (Staudt et al., 2019). For instance, a study by Berman et al. (Berman et al., 2017) found that while three patients with dementia showed cognitive improvements following tPBM treatment, the remaining three did not. Therefore, identifying non-responders at an early stage of the treatment is crucial for developing a more effective tPBM treatment paradigm.

Our previous study was the only investigation to screen non-responders to tPBM among older adults with cognitive decline. In our previous research (Lee K. et al., 2024), aiming at enhancing the cost-effectiveness of tPBM treatment, our group introduced a machine-learning-based approach to classify individuals as responders or non-responders to tPBM using functional near-infrared spectroscopy (fNIRS) data collected prior to the intervention. Although this study was a pioneering effort to identify non-responders using fNIRS, it still had notable limitations. The proposed machine learning model, which used fNIRS data recorded during the recognition memory task (RMT), misclassified responders as non-responders with a probability of 23.81%. This misclassification may lead to the exclusion of potential responders who might benefit from tPBM therapy, necessitating the need for further refinement. In addition, the model achieved an accuracy of only 0.7073 when applied to fNIRS signals recorded during resting state, which is insufficient for practical applications. Considering that the participants were older adults with cognitive decline, performing complex mental tasks to record fNIRS data posed significant challenges. Accordingly, predicting tPBM responsiveness using resting fNIRS signals provides a more convenient and less cognitively demanding alternative (Lin et al., 2018). However, further improvements in the accuracy of resting fNIRS-based prediction of tPBM responsiveness remain necessary.

To address the limitations of our previous approach, this study takes a step further to explore neurophysiological markers to predict the treatment outcomes of tPBM therapy in older adults with cognitive decline based on resting fNIRS data simultaneously recorded during tPBM. Both fNIRS and tPBM share the inherent characteristic of utilizing near-infrared light emitted from a light source, which allows for the investigation of cerebral hemodynamic responses during tPBM therapy using the same device (Kwon and Im, 2020). To achieve this goal, we used newly developed home-based wearable device capable of simultaneously recording fNIRS data and administering tPBM, which allows for continuous monitoring and intervention in a naturalistic setting. Graph theoretical analysis was applied to the resting-state functional networks of hemodynamic responses acquired during initial tPBM sessions to identify new fNIRS biomarkers that can distinguish responders from non-responders. We then investigated whether these biomarkers could effectively screen out non-responders before the intervention was fully completed.

## 2 Materials and methods

### 2.1 Participants

A total of 81 participants aged > 60 years were initially enrolled in the experiment conducted at Inje University Ilsan Paik Hospital. Participants were included if they had concerns regarding cognitive impairment and scored between 20 and 28 on the Korean Mini-Mental State Examination (K-MMSE). Exclusion criteria included a prior history or current diagnosis of severe depression, dementia, or other neurological disorders. Of the total participants, 60 were assigned to the experimental group and received a 12-week tPBM intervention, and the remaining 21 formed the control group and did not undergo any intervention. The control group was recruited to determine whether the improvement in the cognitive function within the experimental group was caused by the tPBM intervention or merely by repeated exposure to cognitive tasks (i.e., the learning effect).

**FIGURE 1:**
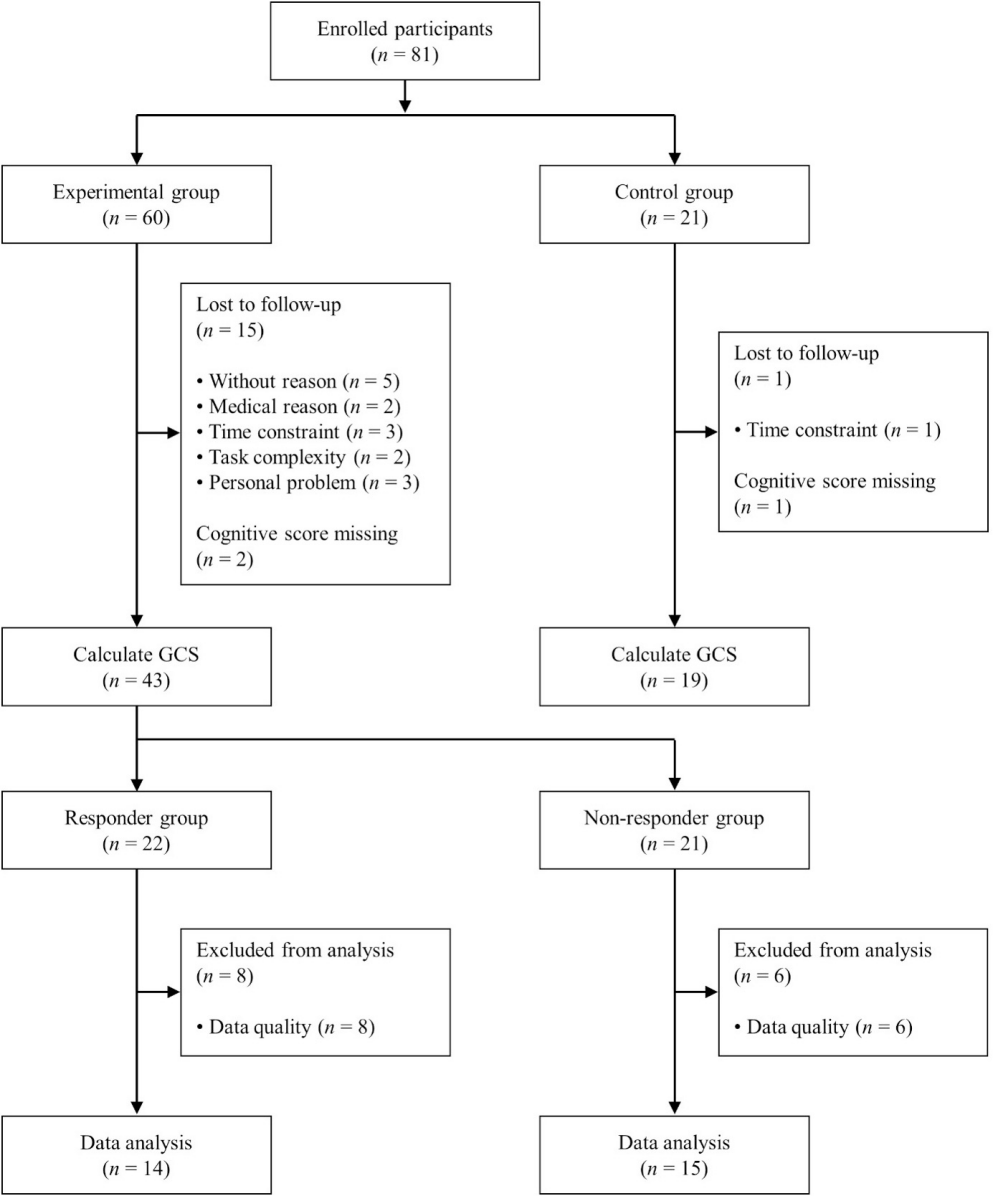
Flowchart depicting participant enrollment, group allocation, and data analysis.

Seventeen and two participants from the experimental and control groups, respectively, were excluded from the analysis owing to missing cognitive test scores or loss to follow-up. Consequently, the cognitive scores acquired from 43 participants in the experimental group and 19 in the control group were used to calculate the global cognitive score (GCS). Among the 43 participants in the experimental group, fNIRS signals from 14 responders and 15 non-responders to tPBM therapy were included in further analyses, whereas 14 datasets were excluded owing to low-quality fNIRS signals (see Supplementary Figure 1). Participants whose fNIRS data from more than five tPBM trials were identified as low quality through visual inspection were excluded from the analysis. Notably, the high exclusion rate was attributed to the fNIRS signals recorded by participants at home. A flowchart detailing the participant exclusion process is presented in Figure 1. All participants provided written informed consent, and the study was approved by the Institutional Review Board of Inje University Ilsan Paik Hospital (IRB no. 2020-11-015).

### 2.2 Wearable device for simultaneously recording fNIRS and administering tPBM

**FIGURE 2:**
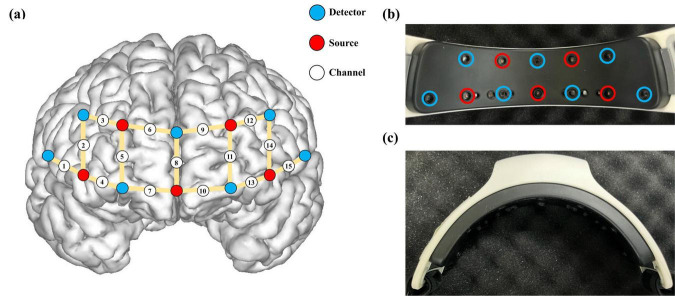
**(a)** Locations of detectors and light sources for the wearable device integrating the fNIRS system with tPBM administration. Photograph showing the **(b)** interior view and **(c)** top-down view of the device.

A custom-made wearable device developed by modifying the light source power and wavelength of a commercial fNIRS device (NIRSIT-LITE; OBELAB Inc., Seoul, South Korea) was used to simultaneously acquire fNIRS data and administer tPBM (Figures 2b,c). Modifications to the tPBM device were made by the original manufacturer, OBELAB, Inc. The device consisted of five light sources and seven photodetectors, each separated by 3 cm, as illustrated in Figure 2a. The light sources emitted infrared light at a power density of 80 mW/cm^2^, utilizing dual wavelengths of 810 and 850 nm. In the tPBM mode, the light was delivered to the brain as a square waveform oscillating at a random frequency within the range of 32–64 Hz with a 50% duty cycle. These parameters were selected based on previous studies demonstrating significant cognitive improvements following tPBM (Zein et al., 2018). In the fNIRS mode, hemodynamic response signals were recorded at a sampling rate of 8.138 Hz from 15 channels in the prefrontal area, as shown in Figure 2a. Notably, fNIRS signals were concurrently recorded during tPBM administration using the dual wavelengths of 810 and 850 nm.

### 2.3 Experimental paradigm

Participants underwent tPBM intervention sessions over a 12-week period and were instructed to remain motionless during stimulation. The frequency of tPBM sessions was determined at the participant’s discretion. Each 15-min session was conducted at home, during which participants watched a silent film while fNIRS data were recorded. The 43 participants in the experimental group adhered to the protocol, each completing at least 20 sessions. Cognitive improvements resulting from the 12-week tPBM intervention were assessed at the Inje University Ilsan Paik Hospital using nine cognitive metrics obtained both before and after the 12-week intervention. These metrics comprised various cognitive domains, including the digit span backward and forward tests (DST-F and DST-B, respectively), verbal fluency task (VFT), three components of the Korean auditory verbal learning test (K-AVLT), Stroop task, digit symbol coding (DSC), and RMT. The detailed paradigms for each cognitive task are provided in Supplementary material.

Participants were categorized as responders or non-responders to tPBM using the method outlined in our previous study (Lee K. et al., 2024). A paired-sample *t*-test was performed within the experimental group to identify cognitive metrics that showed significant improvements following the tPBM intervention. These metrics were then used to calculate the GCS (Gunning et al., 2021), which consolidated multiple cognitive scores into a single composite measure. The GCS scores before and after the 12-week tPBM intervention were defined as the average of the z-score-normalized values, which were calculated based on the mean and standard deviation of each cognitive metric. The participants with a ΔGCS (GCS after the tPBM intervention minus GCS before the tPBM intervention) ≥ 0.5 were classified as responders, while those with a ΔGCS < 0.5 were categorized as non-responders (Gunning et al., 2021; Harvey et al., 2006; Heseltine et al., 1998; McKhann et al., 1997; Norman et al., 2003).

### 2.4 fNIRS preprocessing

The fNIRS data were preprocessed using the BBCI toolbox,^1^ and other in-house scripts were implemented in MATLAB 2023a (MathWorks, Natick, MA, United States). The modified Beer–Lambert law was applied to convert the raw light intensity data into changes in oxy-hemoglobin (ΔHbO) and deoxy-hemoglobin (ΔHbR) concentrations. Because fNIRS signals were acquired in a home setting without strict environmental control, the removal of motion artifacts was critical. To address this issue, motion artifacts were removed by filtering ΔHbO and ΔHbR using wavelet coefficients (Molavi and Dumont, 2012). A sixth-order Butterworth bandpass filter with a passband of 0.01–0.09 Hz was then applied to eliminate physiological and instrumental noise from the signals. For analysis, segments of ΔHbO and ΔHbR that ranged from 10 to 890 s out of the 900 s of recording were selected, as this interval exhibited stable signal characteristics (Duan et al., 2012; Wijeakumar et al., 2012). Baseline correction was performed by subtracting the mean value of each channel in the 5–10 s range from the corresponding segment data. Finally, total-hemoglobin change (ΔHbT) was calculated by summing the ΔHbO and ΔHbR. Because the number of tPBM trials varied among participants and the minimum number of available trials across the 29 participants was 15, all participants were standardized to 15 through random sampling from the entire span.

### 2.5 Functional connectivity analysis

Functional connectivity for HbO, HbR, and HbT was assessed using a simple correlation coefficient analysis between all combinations of two fNIRS channels. To construct the binary matrices, the correlation coefficients exceeding a specific threshold were assigned a value of 1, whereas those below the threshold were assigned a value of 0. Candidate thresholds for binarization ranged from 0.4 to 0.9 in an increment of 0.01 (Chen et al., 2023; Mingming et al., 2024). This process resulted in 15 × 15 adjacency matrices for ΔHbO, ΔHbR, and ΔHbT for all 15 trials and all thresholds. These adjacency matrices were then used for graph theoretical analysis, with each channel representing a node in the network and the binary values (0 or 1) between channel pairs representing the edges connecting the nodes.

To quantify the network properties from adjacency matrices, commonly used graph-theoretical metrics were employed, including degree centrality (DC), global efficiency (GE), and clustering coefficient (CC) (Xu et al., 2015; Yaqub et al., 2018). The DC, which is indicative of the wiring cost of the entire network, was calculated as the total number of edges connected to a node, which is defined as


$$D\,C\,\left(i\right)=\sum_{j\in N}a\,\left(i,j\right)$$

where *a*(*i*,*j*) is the total number of edges connected to node *i* and *N* is the number of nodes.

The GE, which measures the ability to transfer information within the entire brain network, is defined as


$$G\,E\,\left(G\right)=\frac{1}{N\,\left(N-1\right)}\,\sum_{j\neq i\in G}\frac{1}{d\,\left(i,j\right)}$$

where *d*(*i*,*j*) represents the shortest path length between node *i* and node *j* in G.

The CC, a quantitative metric of network interconnectivity, is defined as


$$C\,C\,\left(i\right)=\sum_{i\in N}\frac{2\,L_{i}}{Z_{i}\,\left(Z_{i}-1\right)}$$

where *L_i_* is the number of connected edges between the neighbors of node *i*, and *Z_i_* represents the number of neighbors of node *i*. The graph-theoretical indices were computed using the MATLAB toolbox Graph-theoretic Network Analysis (GRETNA) (Wang et al., 2015). The graph-theoretical indices for the selected 15 tPBM trials were divided into five sequential periods (hereafter denoted as Periods 1–5) and averaged within each period to reduce intra-subject variability in the fNIRS data. The graph-theoretical indices derived from 15 tPBM trials randomly selected from the initial 20 sessions were divided into five sequential periods (hereafter denoted as Periods 1–5) and averaged within each period to reduce intra-subject variability in the fNIRS data. The selection of the initial 20 trials was intended to facilitate early prediction of non-responders, ideally within the first 3 weeks of treatment, considering that the average number of tPBM sessions over the 12-week intervention was 58.19. The mean total duration spanning Periods 1 through 5 was 21.29 ± 7.86 days. Resting-state fNIRS signals are known to exhibit substantial intra- and inter-session variability, particularly under home-based acquisition conditions (Abibullaev et al., 2013; Krauledat et al., 2008). To enhance signal stability and mitigate random fluctuations, graph-theoretical indices were averaged within each three-trial period. This periodic averaging strategy is consistent with prior studies that improved signal reliability by averaging fNIRS features across a set of three task-based trials (Lee K. et al., 2024).

Repeated measures analysis of variance (rmANOVA) was performed to determine the optimal thresholds for each graph-theoretical index. Briefly, each graph-theoretical index for all candidate thresholds was assessed using rmANOVA to evaluate the statistical significance of variations in the index across the five periods between the responders and non-responders. The optimal thresholds for each graph-theoretical index were then identified as the threshold values corresponding to the lowest *p*-values derived from the rmANOVA. Using the optimized graph-theoretical indices, we calculated the variance of indices across periods (denoted by Δperiod 2, Δperiod 3, Δperiod 4, and Δperiod 5) by subtracting the baseline indices (Period 1) from those of Periods 2, 3, 4, and 5. These variances were then linearly regressed with ΔGCS using the MATLAB function “fitlm().” Variances of graph-theoretical indices that showed a significant correlation with GCS were selected as the biomarkers for identifying non-responders. Participants with a predicted ΔGCS value < 0.35 from the simple linear regression were classified as non-responders. The value of 0.35 was empirically determined in this study.

### 2.6 Validation

To validate the generalizability of this predictive model, a leave-one-subject-out cross-validation (LOSO-CV) method was implemented. For each LOSO-CV fold, all optimization and selection steps were conducted using only the training data to prevent data leakage. An rmANOVA was repeatedly performed using only the training data to determine the optimal thresholds for each graph-theoretical index. Subsequently, linear regression models were constructed for the indices that exhibited a correlation with ΔGCS at a threshold of *p* < 0.1 within the training set. In the validation procedure, relaxed threshold of *p* < 0.1 for biomarker selection was adopted to reduce the risk of omitting potentially predictive biomarkers. The ΔGCS of the test participant was then predicted using these pre-established models. In accordance with the established classification protocol, participants with a predicted ΔGCS value below 0.35 were classified as non-responders. A min-max optimization approach was also employed within each training set to objectively determine the optimal classification threshold for non-responder classification. Specifically, the threshold was determined by maximizing the minimum value of sensitivity and specificity, thereby ensuring a balanced performance for identifying both responders and non-responders.

### 2.7 Statistical analysis

Demographic analysis of the responder, non-responder, and control groups was conducted using the chi-square test to evaluate sex differences and the Kruskal–Wallis test to assess differences in MMSE scores, educational levels, and age, because the demographic data did not exhibit normal distribution. The Kruskal–Wallis test was also used to evaluate the difference in ΔGCS among the responder, non-responder, and control groups. Subsequently, the Bonferroni-corrected Wilcoxon rank-sum test was conducted for *post hoc* analysis. For the demographic analysis between the responder and non-responder groups included in the fNIRS analysis, the difference in sex composition was assessed using the chi-square test, and other demographic information was analyzed using the Wilcoxon rank-sum test. To account for multiple comparisons and ensure the robustness of the observed correlations, a one-tailed max-*t* permutation test was performed with 10,000 iterations using the correlation coefficients. For this analysis, the five measurement periods were determined as a single family to control the family-wise error rate.

## 3 Results

### 3.1 Demographic comparison and session count analysis

The results for the categorization of responder and non-responder groups were similar to those in our previous study, and the detailed results are presented in Supplementary Table 1. Of the 43 participants in the experimental group who successfully completed the 12-week tPBM intervention, 22 were classified as responders and 21 as non-responders. Data from 43 participants were used for the evaluation of GCS scores, but fNIRS signals only from 14 responders and 15 non-responders to tPBM therapy were used for further prediction studies, as previously mentioned. The detailed demographic information of the 43 participants is presented in Supplementary Table 2. No statistically significant differences were observed in demographic characteristics, except for ΔGCS, which exhibited a significant difference (*p* < 0.001) among the responder, non-responder, and control groups. The responder group showed statistically higher ΔGCS than those of the non-responder and control groups (Bonferroni-corrected *p* < 0.001 for both groups) (see Supplementary Figure 2). The non-responder group did not show a significant difference in ΔGCS from that of the control group (Bonferroni-corrected *p* = 0.264).

**FIGURE 3:**
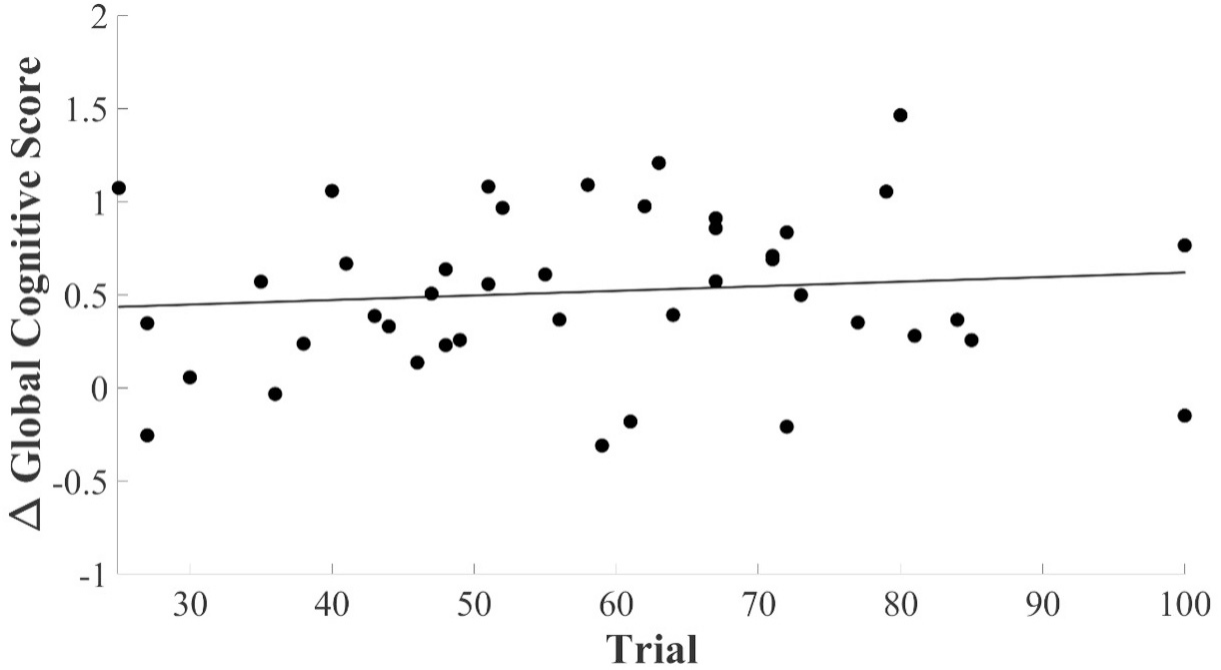
Scatter plot showing the relationship between the number of tPBM trials and ΔGCS. Each data point represents an individual participant, and the line indicates the linear regression trend. No significant correlation was observed between ΔGCS and the number of trials (*p* = 0.4923).

To identify whether the number of tPBM intervention sessions impacted responsiveness to tPBM, we performed a linear regression analysis between ΔGCS and the number of tPBM intervention sessions. The analysis revealed no correlation between ΔGCS and the number of tPBM trials (mean = 58.19 ± 18.88) in the experimental group (*p* = 0.492), as shown in Figure 3. These results not only indicate that the number of tPBM sessions did not influence the categorization of tPBM responsiveness in this study but also confirm that responsiveness can be predicted independently of the total number of sessions using fNIRS data acquired during the initial 20 tPBM sessions.

### 3.2 Correlation between graph-theoretical indices and ΔGCS

After fNIRS preprocessing, data from 14 responders and 15 non-responders were analyzed. As shown in Table 1, individuals in the responder non-responder groups who were included in the analysis did not significantly differ in age, sex, education, K-MMSE score, and pre-tPBM GCS. The thresholds for calculating the CC of ΔHbO, ΔHbR, and ΔHbT derived from rmANOVA are 0.54 [*F*(4, 28) = 3.3312, *p* = 0.013], 0.58 [*F*(4, 28] = 3.4088, *p* = 0.012], and 0.78 [*F*(4, 28) = 2.6209, *p* = 0.039], respectively. The thresholds of the DC are 0.69 [*F*(4, 28) = 2.4251, *p* = 0.052], 0.65 [*F*(4, 28) = 3.7324, *p* = 0.007], and 0.4 [*F*(4, 28) = 2.1519, *p* = 0.079], for ΔHbO, ΔHbR, and ΔHbT, respectively. The thresholds of the GE are 0.79 [*F*(4, 28) = 2.3486, *p* = 0.066], 0.76 [*F*(4, 28) = 3.2481, *p* = 0.015], and 0.67 [*F*(4, 28) = 2.0222, *p* = 0.096] for ΔHbO, ΔHbR, and ΔHbT, respectively. The *R*^2^ and *p*-value of correlation between graph-theoretical indices of baseline and each Δperiod and ΔGCS are presented in Table 2.

**TABLE 1:** Demographic information of the responder and non-responder groups included in the fNIRS analysis.

Characteristics	Responder	Non-responder
***N***	14	15
Male/Female	3/11	7/8
*p*	0.153	
**Age (years)**		
Mean	71	72.6
(SD)	(3.88)	(4.37)
*p*	0.2915	
**Education**		
Mean	9.93	12.2
(SD)	(4.51)	(4.11)
*p*	0.1654	
**K-MMSE**		
Mean	27.36	26.47
(SD)	(1.34)	(1.96)
*p*	0.064	
**Pre-tPBM score**		
Mean	–0.04	–0.04
(SD)	(0.29)	(0.73)
*p*	0.9478	

**TABLE 2:** *R*^2^ and *p*-value of correlation analysis between graph-theoretical indices and ΔGCS.

Graph-theoretical indices		Baseline	Δ Period 2	Δ Period 3	Δ Period 4	Δ Period 5
ΔHbO	**CC**					
	*R* ^2^	0.0261	0.0037	0.0274	0.2286	0.1737
	*p*	0.4025	0.7547	0.3911	**0.0087	*0.0245
	**DC**					
	*R* ^2^	0.0058	0.0061	0.0160	0.1088	0.1625
	*p*	0.6948	0.6861	0.5138	0.0806	*0.0302
	**GE**					
	*R* ^2^	0.0023	0.0049	0.0021	0.0794	0.1267
	*p*	0.8046	0.7195	0.8149	0.1387	0.0581
ΔHbR	**CC**					
	*R* ^2^	0.0344	6.7457 × 10^–6^	3.2768 × 10^–4^	0.1944	0.1667
	*p*	0.3352	0.9893	0.9257	*0.0167	*0.0279
	**DC**					
	*R* ^2^	0.0006	0.0237	0.0271	0.0432	0.0625
	*p*	0.8976	0.4248	0.3936	0.2791	0.1907
	**GE**					
	*R* ^2^	0.0064	0.0436	0.0416	0.0017	0.0072
	*p*	0.6801	0.2771	0.2886	0.8325	0.6616
ΔHbT	**CC**					
	*R* ^2^	0.0166	1.5238 × 10^–6^	0.0714	0.1296	0.1064
	*P*	0.5059	0.9949	0.1612	0.0551	0.0841
	**DC**					
	*R* ^2^	0.0061	0.0022	0.1075	0.0314	0.0013
	*p*	0.6868	0.8108	0.0825	0.3576	0.8516
	**GE**					
	*R* ^2^	0.0080	0.0024	0.1596	0.0388	0.0061
	*p*	0.6441	0.7997	*0.0318	0.3055	0.6866

CC, DC, GE indicates the clustering coefficient, degree centrality, and global efficiency, respectively. Significance levels are marked as follows: *p < 0.05 and **p < 0.01.

### 3.3 Classification results

**FIGURE 4:**
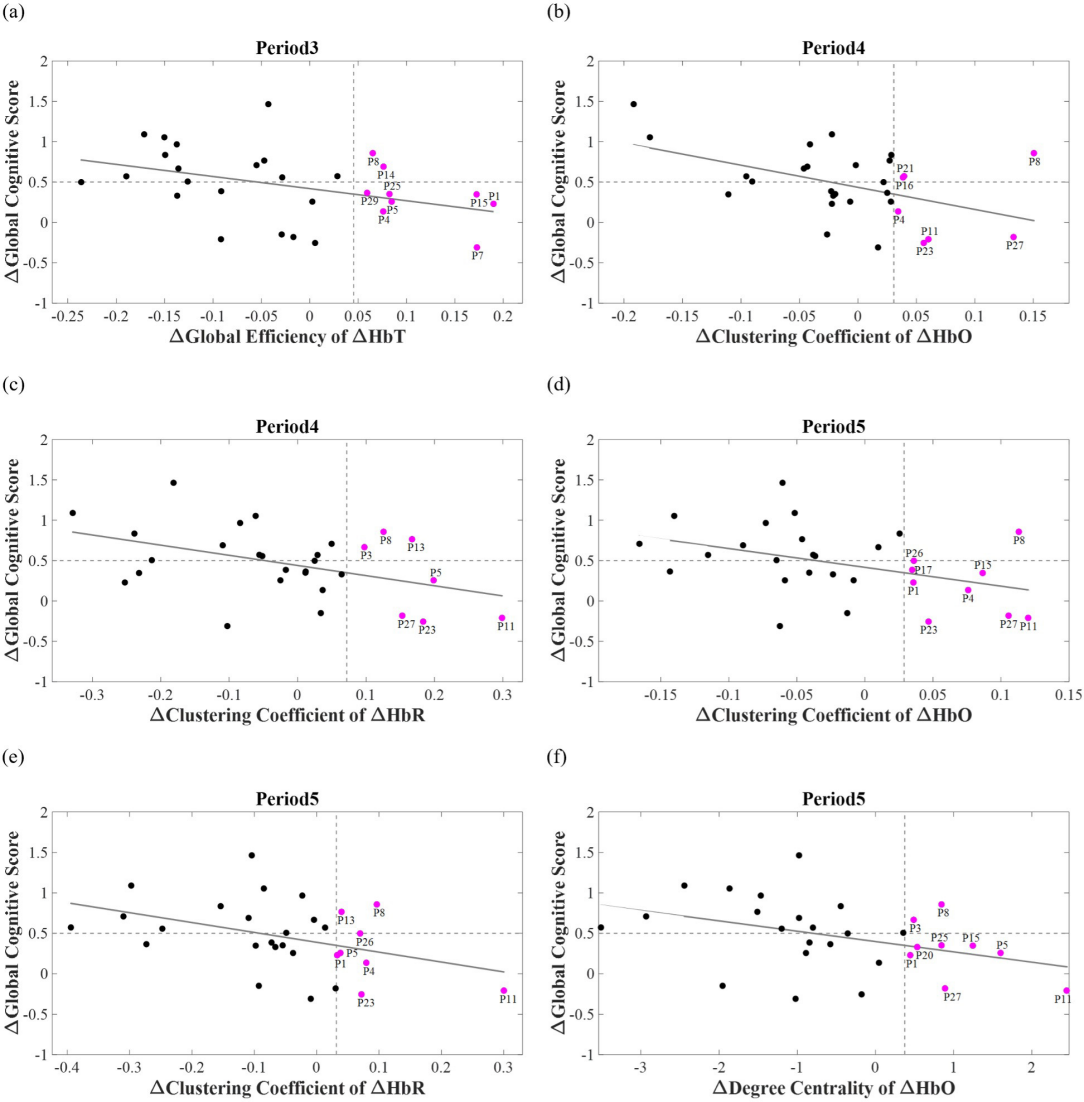
Correlation between ΔGCS and changes in: **(a)** global efficiency of ΔHbT at period 3, **(b)** clustering coefficient of ΔHbO at period 4, **(c)** clustering coefficient of ΔHbR at period 4, **(d)** clustering coefficient of ΔHbO at period 5, **(e)** clustering coefficient of ΔHbR at period 5, and **(f)** degree centrality of ΔHbO at period 5. The vertical dashed line indicates the prediction criteria for non-responders, whereas the horizontal dashed line represents the ΔGCS threshold of 0.5, which is used to categorize participants as responders and non-responders. Magenta dots indicate participants predicted as non-responders, with participant indices displayed adjacent to the magenta dots. Notably, those located in the lower-right quadrant correspond to participants correctly identified as non-responders.

**FIGURE 5:**
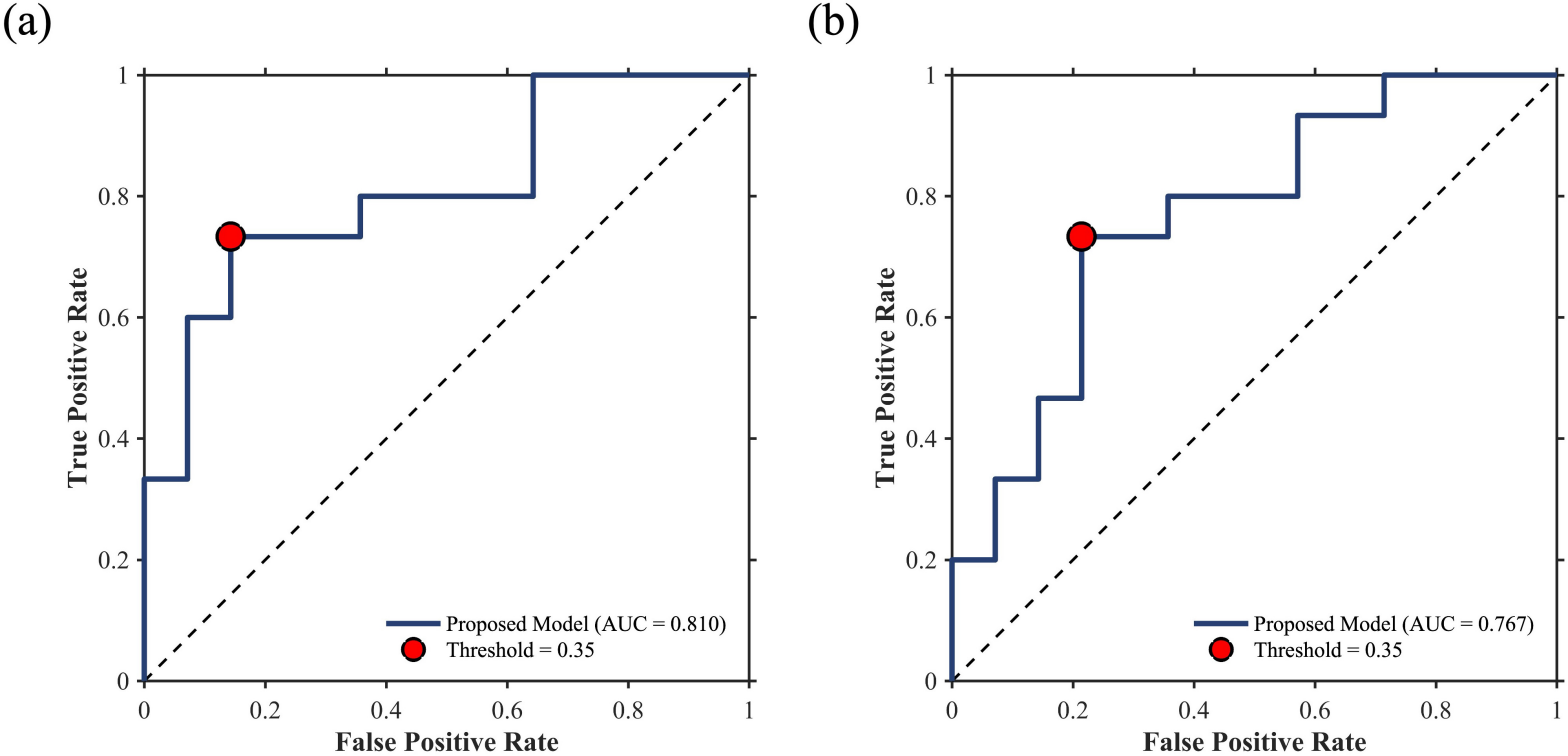
ROC curves for non-responder prediction. **(a)** Whole-dataset performance (AUC = 0.810) and **(b)** LOSO-CV validation performance (AUC = 0.767). Red dots indicate the classification performance at the threshold of 0.35.

The graph-theoretical indices of baseline and Δperiod 2 did not correlate with ΔGCS. The GE of the ΔHbT in Δperiod 3 correlated with ΔGCS (*R*^2^ = 0.1596, *p* < 0.05). In period 3, nine participants were postulated to be non-responders, with a threshold of 0.35. Of these, seven did not exhibit an actual response to the tPBM sessions, whereas the remaining two were affiliated with the responder group. In period 4, the CC of HbO (*R*^2^ = 0.2286, *p* < 0.01) and HbR (*R*^2^ = 0.1944, *p* < 0.05) correlated with ΔGCS. A hard voting mechanism was employed using two graph-theoretical indices—CCs of ΔHbO and ΔHbR, which showed significance—to classify non-responders in Period 4. This led to the categorization of four participants as non-responders, including one who was previously misclassified as a non-responder. In Δperiod 5, three graph-theoretical indices correlated with ΔGCS—CCs of ΔHbO (*R*^2^ = 0.1737, *p* < 0.05) and ΔHbR (*R*^2^ = 0.1667, *p* < 0.05), and DC of ΔHbO (*R*^2^ = 0.1625, *p* < 0.05). In Period 5, three graph-theoretical indices were used to classify non-responders using a majority voting scheme. Nine participants were postulated as non-responders, and one of them, who had been misclassified in periods 3 and 4, was included. With eight participants not showing an actual response, four and two overlapped with those classified as non-responders in Periods 3 and 4, respectively. As a result, 13 participants were identified as non-responders, with 11 verified as non-responders through the tPBM interventions (see Figure 4). As shown in Figure 5a, a Receiver Operating Characteristic (ROC) curve analysis demonstrated a high predictive accuracy with an Area Under the Curve (AUC) of 0.810. Specifically, the threshold of 0.35 was identified as the optimal cut-off point with an accuracy of 79.31%, a sensitivity of 73.33% in identifying non-responders, a specificity of 85.71%, a positive predictive value (PPV) of 78.67%, and a negative predictive value (NPV) of 75.00%.

### 3.4 Validation results

**FIGURE 6:**
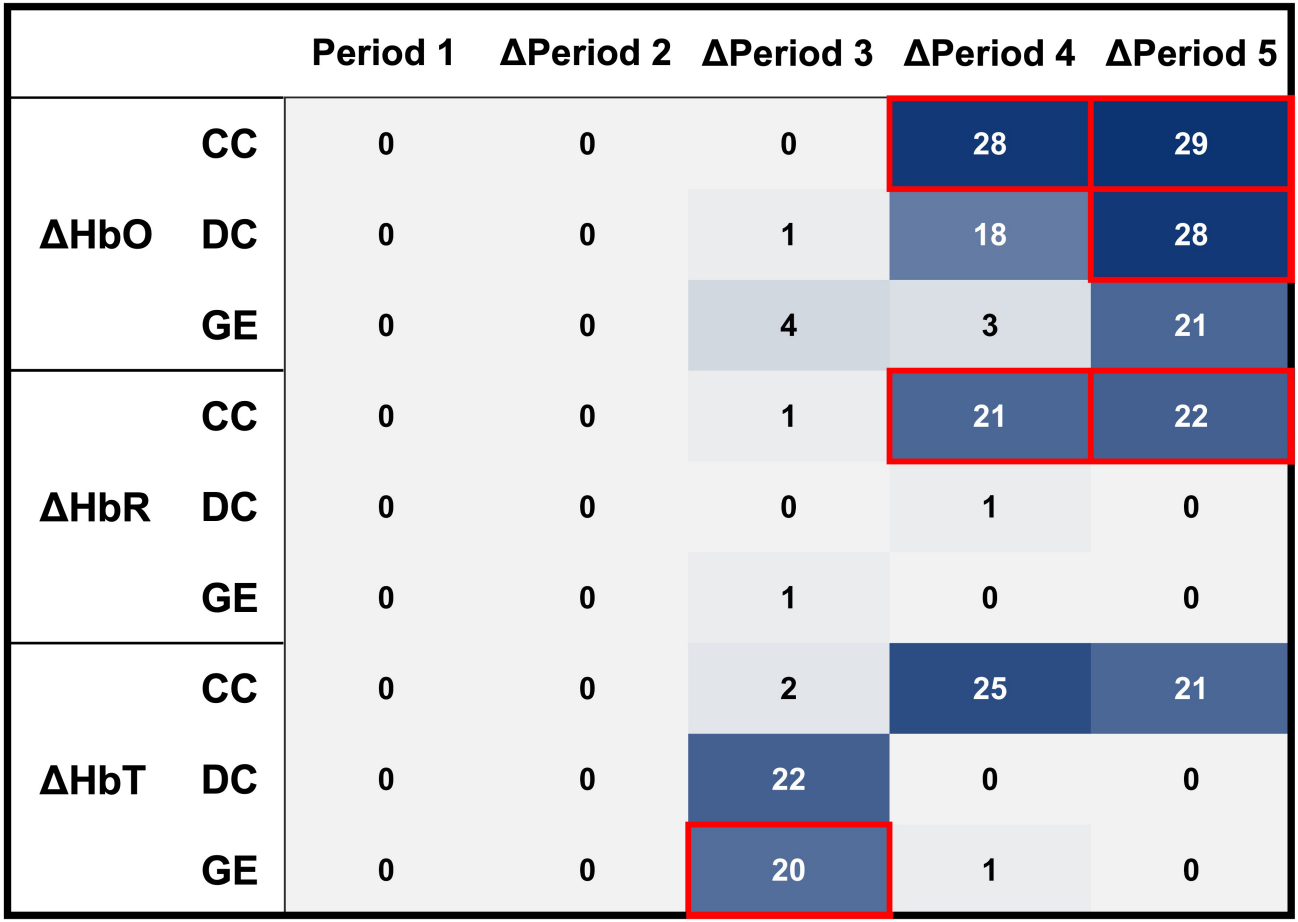
Selection frequency of each graph-theoretical index across 29 LOSO-CV folds. The red boxes highlight the key biomarkers identified for the prediction of non-responders.

Table 3 summarizes the mean binarization thresholds for each graph-theoretical index calculated during the LOSO-CV procedure. These derived thresholds remained highly consistent across folds, demonstrating an average root-mean-square error of 0.0608 compared to the whole-dataset thresholds. During the LOSO-CV process, an identical classification protocol was employed. Specifically, when three or fewer biomarkers were selected, the aforementioned voting mechanism was applied. If the number of selected biomarkers exceeded three, the test participant was classified as a non-responder if at least two biomarkers indicated non-responsiveness. This strategy was intentionally adopted to prioritize model sensitivity in predicting potential non-responders. Consequently, at a classification threshold of 0.35, the predictive model achieved an accuracy of 75.86%, a sensitivity of 73.33%, a specificity of 78.57%, a PPV of 78.57%, and a NPV of 73.33%. The ROC curve for non-responder prediction during the LOSO-CV process is presented in Figure 5b, yielding an AUC of 0.767. The selection frequency of each graph-theoretical index across the LOSO-CV folds is illustrated in Figure 6. Furthermore, min-max optimization of the classification threshold across the LOSO-CV folds yielded consistent performance, with an optimal mean threshold of 0.3472 ± 0.0225, which is closely aligned with the initial threshold of 0.35.

**TABLE 3:** Optimal binarization thresholds for graph-theoretical indices across whole-dataset and LOSO-CV analyses.

Graph-theoretical indices	Δ HbO			Δ HbR			Δ HbT		
	CC	DC	GE	CC	DC	GE	CC	DC	GE
Whole-dataset	0.54	0.58	0.78	0.69	0.65	0.4	0.79	0.76	0.67
LOSO-CV	0.5486 ± 0.0464	0.5955 ± 0.0220	0.7525 ± 0.0965	0.6786 ± 0.0432	0.6500 ± 0.0000	0.4272 ± 0.0832	0.7341 ± 0.0962	0.7472 ± 0.0339	0.6328 ± 0.0947

## 4 Discussion

In this study, we attempted to predict tPBM non-responders in the early stages of the tPBM intervention using graph-theoretical indices of the prefrontal functional connectivity network estimated with resting fNIRS signals simultaneously recorded during tPBM administration. To the best of our knowledge, this is the first study to show that tPBM responsiveness can be predicted at an early stage of the intervention using resting fNIRS signals concurrently recorded during the intervention. Furthermore, this study demonstrates the potential of a device integrating fNIRS and tPBM in a single, wearable platform, enabling continuous monitoring of cerebral hemodynamic responses during tPBM therapy. Each graph-theoretical index of each period was analyzed using the correlation coefficient with the ΔGCS. The changes in the GE of ΔHbT in Period 3, CC of ΔHbO and ΔHbR in Period 4, and CCs of ΔHbO and ΔHbR and DC of ΔHbO in Period 5 had significant negative correlations with ΔGCS. Using these graph-theoretical indices, linear regression was performed for the prediction of non-responders. Those whose regression results were below the threshold of 0.35 were classified as non-responders to tPBM. As a result, of the 13 participants who were predicted to be non-responders, 11 were confirmed to be actual non-responders after the 12-week tPBM intervention. Furthermore, implementation of LOSO-CV verified the generalizability of the predictive model, maintaining a sensitivity of 73.33%.

For the tPBM-fNIRS hybrid system used in this study, the device was custom-modified from the commercially available NIRSIT LITE system (originally configured with 780 nm/850 nm dual-wavelength light sources). The modified system adopted a dual-wavelength configuration of 810 and 850 nm, with an increased light source power density of 80 mW/cm^2^. This adjustment was motivated by previous studies demonstrating *in vivo* efficacy of 810 nm tPBM at an energy density of 36 J/cm^2^ (Ando et al., 2011; Wu et al., 2012; Zein et al., 2018; Zhang et al., 2014). To replicate this condition, we applied 810 nm stimulation with a 50% duty cycle over 15 min, which allowed the delivery of the equivalent energy density (36 J/cm^2^). As a result, improvements were observed in seven out of nine cognitive performance metrics after the 12-week intervention. Importantly, the selected parameters remain within the safety guidelines recommended for fNIRS instrumentation. While light power density is a critical safety concern in wearable systems, prior fNIRS studies have safely operated at power densities below 200 mW/cm^2^ (Chenier and Sawan, 2007; Sanfilippo et al., 2014). Moreover, although the use of 810 nm may theoretically introduce a slightly higher degree of spectral crosstalk compared to 780 nm, it remains one of the commonly employed wavelengths in fNIRS systems (Saikia et al., 2019; Wyser et al., 2017). Notably, previous optical modeling studies have identified the optimal fitting windows for deoxy-hemoglobin and oxy-hemoglobin concentration estimation to be 720–810 nm and 760–860 nm (Hashem et al., 2021), respectively. Based on these findings, the application of 810 nm in this study can be regarded as feasible for both hemodynamic monitoring and tPBM, despite the potential trade-off in crosstalk.

To rigorously verify that the observed negative correlations were not driven by random chance, a one-tailed max-*t* permutation test was employed to account for multiple comparisons. Considering the physiological interdependence between ΔHbO, ΔHbR, and ΔHbT, as well as the fact that graph-theoretical indices are derived from the same adjacency matrix, these variables cannot be treated as strictly independent. Therefore, we grouped these indices across the five measurement periods into a single family to strictly control the family-wise error rate. Notably, under the permutation-based correction, the correlation between ΔGCS and CC of ΔHbO and ΔHbR in Period 4 remained statistically significant (*p* < 0.05). Furthermore, other biomarkers including GE of ΔHbT in Period 3 (*p* = 0.0774), CC of ΔHbO and ΔHbR in Period 5 (*p* = 0.0616 and *p* = 0.0731, respectively), and DC of ΔHbO in Period 5 (*p* = 0.0749) showed a consistent negative trend with ΔGCS. The reliability of these proposed biomarkers is further supported by the selection frequency during the LOSO-CV procedure. As shown in Figure 6, these features were consistently identified as key predictors across independent validation folds, underscoring their generalizability and potential as stable clinical biomarkers.

The negative correlation between the changes in graph-theoretical indices and ΔGCS in this study suggests that an improvement in cognitive ability is associated with a decrease in functional connectivity. Correlations between brain networks and cognitive abilities have been previously demonstrated. Specifically, a previous fNIRS study demonstrated increased resting-state functional connectivity within the prefrontal cortex in patients with MCI compared with that of HCs (Nguyen et al., 2019). Additionally, an fMRI study showed that the efficiency and CC of the SCD group were significantly higher than those of HCs in the frontal region during the resting state (Chen H. et al., 2020). Furthermore, patients with MCI showed a significantly higher DC and efficiency in the frontal region than in HCs (Luo et al., 2021). These enhanced prefrontal functional connectivity characteristics in older adults with cognitive decline may reflect a compensatory mechanism for reduced networks between areas (Chen H. et al., 2020; Luo et al., 2021; Nguyen et al., 2019; Wang et al., 2007). Consequently, the decrease in the graph-theoretical indices of responders may indicate a disruption of the compensatory mechanism due to tPBM therapy. This decrease in graph-theoretical indices may be observed in the responder group even after the first few tPBM sessions, suggesting that these measures could effectively predict tPBM responsiveness.

In this study, graph theoretical indices potentially predicted the efficacy of tPBM therapy. The effectiveness of tPBM therapy in older adults could be evaluated with the changes in ΔHbO during the cognitive task after tPBM stimulation (Chan et al., 2021; Lee and Chan, 2023). Additionally, the averaged ΔHbO values were the dominant feature from our previous study based on machine learning (Lee K. et al., 2024). By aggregating the results of previous studies, we performed a correlation analysis between ΔHbO and ΔGCS, consistent with the analysis conducted using graph-theoretical indices. However, no significant correlation was observed in this study between the averaged ΔHbO in Period 1 (*R*^2^ = 0.0068, *p* = 0.671) and changes of averaged ΔHbO in Periods 2 (*R*^2^ = 0.0102, *p* = 0.603), 3 (*R*^2^ = 0.0573, *p* = 0.211), 4 (*R*^2^ = 0.0240, *p* = 0.422), and 5 (*R*^2^ = 0.0274, *p* = 0.391). These outcomes could be attributed to the fact that previous studies recorded fNIRS signals during cognitive tasks conducted after tPBM sessions, whereas our study used fNIRS signals recorded during stimulation without any specific cognitive tasks.

Our study has some limitations. First, recruitment challenges and a rigorous exclusion of contaminated home-based fNIRS data resulted in a relatively small sample size, potentially restricting the generalizability of our findings. However, statistical comparisons between the included participants and those excluded due to signal artifacts revealed no significant differences in demographic information (see Supplementary Table 3). These results suggest that our final dataset was not significantly affected by selection bias and remained representative of the initial cohort. Second, this study lacked a sham group to account for placebo effects. However, this study primarily focused on identifying neurophysiological markers that characterize individual treatment responsiveness within an intervention group. This approach, which prioritizes the prediction of personalized responsiveness rather than absolute efficacy, aligns with other clinical studies predicting individualized treatment responses (Cui et al., 2019; Ho et al., 2025). Moreover, the number of tPBM sessions varied among patients because of the home-based treatment setting. Although no correlation was observed between tPBM session frequency and ΔGCS, this uncontrolled factor may still have influenced the results. Nevertheless, we identified statistically significant indices that appears capable of differentiating the therapeutic efficacy of tPBM in older adults with cognitive decline. Furthermore, the ability to predict tPBM responsiveness using resting-state fNIRS offers an opportunity to adaptively adjust stimulation parameters—such as intensity or duration—if a patient is predicted to be a non-responder. This adaptive treatment strategy may herald a new paradigm for managing cognitive impairment in the elderly population.

## Data Availability

The datasets presented in this article are not readily available because data cannot be shared according to the IRB approval. Requests to access the datasets should be directed to Chang-Hwan Im, ich@hanyang.ac.kr.

## Footnotes

1
https://github.com/bbci/bbci_public
