# Supplementary material for: Prediction of treatment responsiveness to home-based transcranial photobiomodulation (tPBM) intervention for cognitive decline using fNIRS concurrently recorded during tPBM
Source: Front Aging Neurosci. 2026 Feb 12;18:1716502. doi: 10.3389/fnagi.2026.1716502 (PMC12936009; doi:10.3389/fnagi.2026.1716502)
Supplement: Supplementary file 1 [file Table_1.docx]

Supplementary Material

# Experimental Paradigm

The digit span task (DST) is employed to assess attention, concentration, and working memory. Participants are required to memorize provided digits in either a forward or backward sequence. The number of digits to be remembered increases with each trial, commencing with three digits initially.

The Korean adult verbal learning test (K-AVLT) is a cognitive task involving the memorization of a series of words. K-AVLT consists of five immediate recall trials, a delayed recall trial, and a recognition trial. The verbal memory is evaluated with this task.

The digit symbol coding (DSC) test comprises nine symbols corresponding to numbers 1-9. Participants are required to match these symbols to the randomly arranged numbers quickly and accurately. DSC serves as an assessment tool for evaluating working memory, attention, motor speed, and visuoperceptual functions.

The Stroop task comprises two conditions which is the color naming condition and the word naming condition. In the color naming condition, participants are required to identify font color rather than the meaning of word. Conversely, in the word naming condition, the meaning of word in the target. Each condition consists of two sessions, each with 12 trials. This task measures the executive function of participants.

The recognition memory task (RMT) consists of two phases, encoding and recognition. In the encoding phase, participants are tasked with memorizing 15 words within 3 minutes. In the subsequent recognition phase, 30 words are presented, encompassing the original 15 words from the encoding phase and an additional 15 lure words, all randomly displayed to the participants. Participants are then tasked with determining whether each word was included in the encoding phase or not. RMT is employed to assess memory function.

The verbal fluency test (VFT) is cognitive task in which participants are instructed to generate as many words as possible that begin with the Korean letter ‘ㄱ’ and ‘ㅅ’ which phonetically correspond to the English letters ‘k’ and ‘s’, respectively. The VFT measures verbal proficiency and executive control.

# Supplementary Figures and Tables

## Supplementary Tables

**Supplementary Table 1.** The baseline scores and post-tPBM scores of tPBM group.

|  | **Pre-tPBM score** | **Post-tPBM score** | ***p*** |
| --- | --- | --- | --- |
| **VFT** | 11.58 ± 4.91 | 13.49 ± 4.07 | ** 0.0067 |
| **K-AVLT (Immediate)** | 36.3 ± 9.26 | 41.72 ± 10.51 | *** 1.5917×10^-4^ |
| **K-AVLT (Delayed)** | 6.88 ± 2.95 | 8.53 ± 3.4 | *** 5.4910×10^-4^ |
| **K-AVLT (Recognition)** | 12.4 ± 1.75 | 13.49 ± 1.75 | *** 4.9815×10^-5^ |
| **DST-F** | 9.09 ± 2.11 | 10.11 ± 2.15 | ** 0.0016 |
| **DST-B** | 7.26 ± 2.34 | 7.14 ± 2.11 | 0.6626 |
| **DSC** | 43.35 ± 15.39 | 44.93 ± 13.45 | 0.2519 |
| **Stroop task** | 0.48 ± 0.18 | 0.6 ± 0.16 | *** 3.4408×10^-5^ |
| **RMT** | 0.44 ± 0.13 | 0.49 ± 0.11 | ** 0.0091 |

*p<0.05, **p<0.01, and ***p<0.001

**Supplementary Table 2.** Demographic information of the responder, non-responder, and control groups

|  | **Responder** | **Non-responder** | **Control** |
| --- | --- | --- | --- |
| **N** | 22 | 21 | 19 |
| Male / Female | 4 / 18 | 7 / 14 | 10 / 9 |
| *p* | 0.067 |  |  |
| **Age (years)** |  |  |  |
| Mean | 70.41 | 72.24 | 71.11 |
| (SD) | (4.4) | (4.55) | (5.23) |
| *p* | 0.4036 |  |  |
| **Education** |  |  |  |
| Mean | 9.55 | 11.81 | 12.05 |
| (SD) | (4.08) | (3.78) | (4.26) |
| *p* | 0.0704 |  |  |
| **K-MMSE score** |  |  |  |
| Mean | 26.91 | 26.67 | 26.47 |
| (SD) | (1.97) | (1.74) | (2.22) |
| *p* | 0.5553 |  |  |
| **Pre-tPBM score** |  |  |  |
| Mean | –0.24 | 0.07 | 0.21 |
| (SD) | (0.49) | (0.7) | (0.72) |
| *p* | 0.0892 |  |  |

**Supplementary Table 3.** Demographic information of the responder, non-responder, and control groups

|  | **Included participants** | **Excluded participants** |
| --- | --- | --- |
| **N** | 29 | 14 |
| Male / Female | 10 / 19 | 1 / 13 |
| *p* | 0.0710 |  |
| **Age (years)** |  |  |
| Mean | 71.83 | 70.21 |
| (SD) | (4.15) | (5.19) |
| *p* | 0.2144 |  |
| **Education** |  |  |
| Mean | 11.10 | 9.71 |
| (SD) | (4.39) | (3.20) |
| *p* | 0.3138 |  |
| **K-MMSE** |  |  |
| Mean | 26.90 | 26.57 |
| (SD) | (1.72) | (2.14) |
| *p* | 0.6033 |  |
| **Pre-tPBM score** |  |  |
| Mean | –0.04 | –0.20 |
| (SD) | (0.55) | (0.73) |
| *p* | 0.3853 |  |

## Supplementary Figures
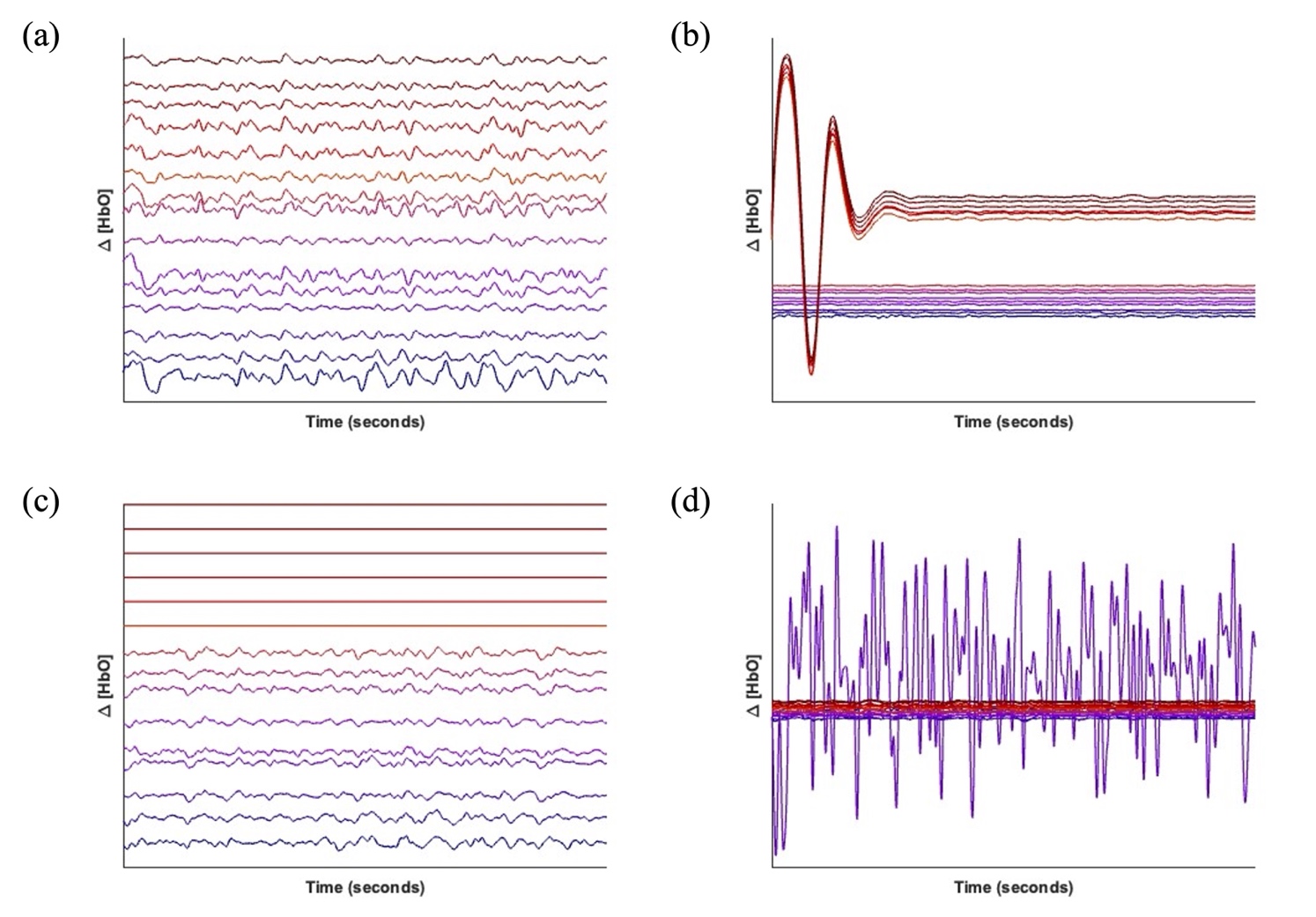


**Supplementary Figure 1.** Representative fNIRS time-series data illustrating signal quality control. (a) Acceptable ΔHbO signals showing typical hemodynamic oscillations. (b – d) Examples of excluded signals due to technical issues and artifacts.


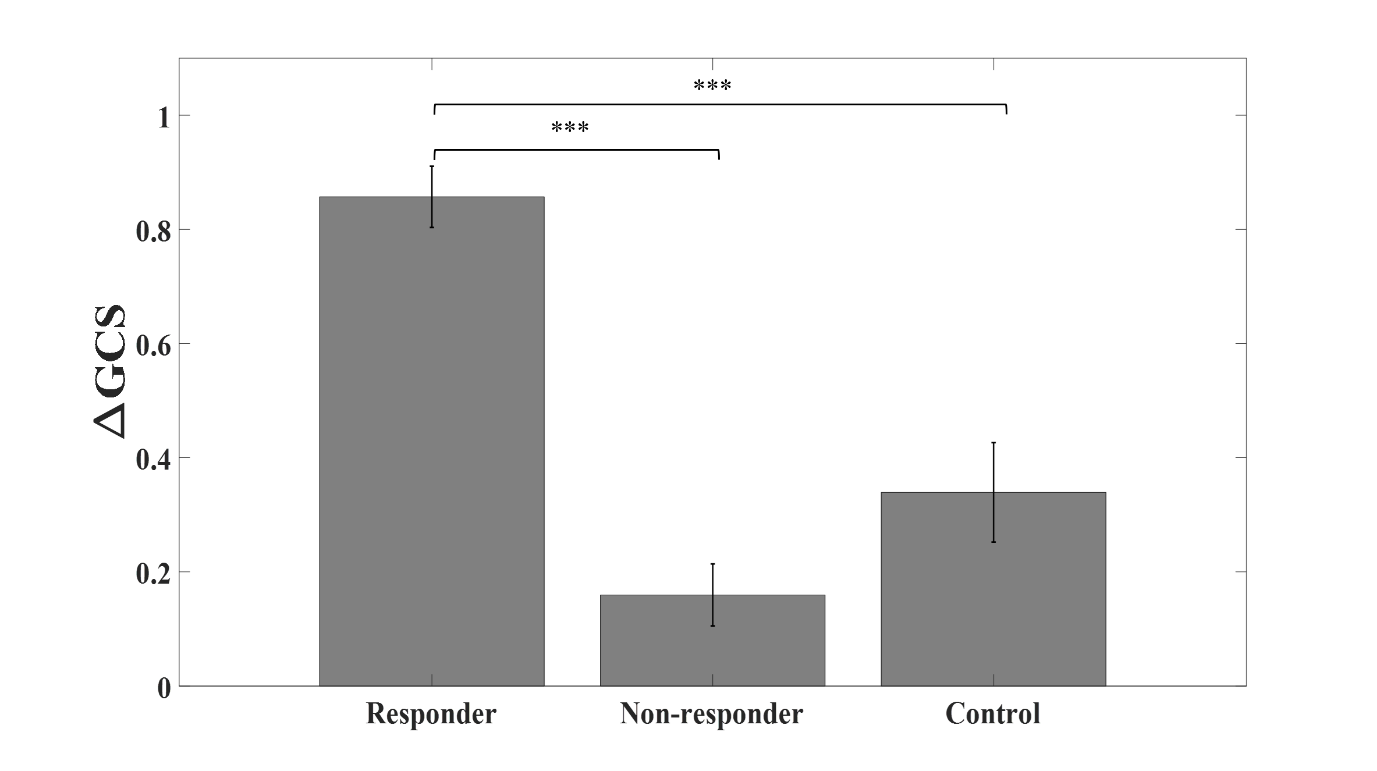


**Supplementary Figure 2.** ΔGCS of responder, non-responder, and control groups.
